# Pre-Clinical Development of a Humanized Anti-CD47 Antibody with Anti-Cancer Therapeutic Potential

**DOI:** 10.1371/journal.pone.0137345

**Published:** 2015-09-21

**Authors:** Jie Liu, Lijuan Wang, Feifei Zhao, Serena Tseng, Cyndhavi Narayanan, Lei Shura, Stephen Willingham, Maureen Howard, Susan Prohaska, Jens Volkmer, Mark Chao, Irving L. Weissman, Ravindra Majeti

**Affiliations:** 1 Institute for Stem Cell Biology and Regenerative Medicine and the Ludwig Cancer Center, Stanford University School of Medicine, Stanford, California, United States of America; 2 Department of Medicine, Division of Hematology, Stanford University School of Medicine, Stanford, California, United States of America; Emory University, UNITED STATES

## Abstract

CD47 is a widely expressed cell surface protein that functions as a regulator of phagocytosis mediated by cells of the innate immune system, such as macrophages and dendritic cells. CD47 serves as the ligand for a receptor on these innate immune cells, SIRP-alpha, which in turn delivers an inhibitory signal for phagocytosis. We previously found increased expression of CD47 on primary human acute myeloid leukemia (AML) stem cells, and demonstrated that blocking monoclonal antibodies directed against CD47 enabled the phagocytosis and elimination of AML, non-Hodgkin’s lymphoma (NHL), and many solid tumors in xenograft models. Here, we report the development of a humanized anti-CD47 antibody with potent efficacy and favorable toxicokinetic properties as a candidate therapeutic. A novel monoclonal anti-human CD47 antibody, 5F9, was generated, and antibody humanization was carried out by grafting its complementarity determining regions (CDRs) onto a human IgG4 format. The resulting humanized 5F9 antibody (Hu5F9-G4) bound monomeric human CD47 with an 8 nM affinity. Hu5F9-G4 induced potent macrophage-mediated phagocytosis of primary human AML cells in vitro and completely eradicated human AML in vivo, leading to long-term disease-free survival of patient-derived xenografts. Moreover, Hu5F9-G4 synergized with rituximab to eliminate NHL engraftment and cure xenografted mice. Finally, toxicokinetic studies in non-human primates showed that Hu5F9-G4 could be safely administered intravenously at doses able to achieve potentially therapeutic serum levels. Thus, Hu5F9-G4 is actively being developed for and has been entered into clinical trials in patients with AML and solid tumors (ClinicalTrials.gov identifier: NCT02216409).

## Introduction

The development of cancer requires normal cells to acquire methods to dysregulate proliferation, avoid programmed cell death, and acquire many of the other hallmarks of cancer [1]. In addition, cancer cells must evade programmed cell removal, which is the phagocytic elimination of aberrant cells by cells of the innate immune system including macrophages, dendritic cells, and neutrophils [2]. The stimulation of programmed cell removal utilizes a number of pro-phagocytic signals, many of which are not molecularly characterized, but can include protein signals such as calreticulin [3], phospholipids such as phosphatidylserine, and abnormal glycosylation. However, the inhibition of programmed cell removal is primarily inhibited by a single dominant molecule, CD47. All human cancers studied to date, including both solid tumors and leukemia, express CD47, making CD47 a universal target in human cancer.

Human acute myeloid leukemia (AML) is an aggressive malignancy of bone marrow progenitors, characterized by an increase in immature white blood cells and bone marrow failure. AML is the most common type of acute leukemia affecting adults, with a poor prognosis and few therapeutic options. Current standard of care for medically fit AML patients consists of high dose chemotherapy, often including allogeneic hematopoietic cell transplantation. Even with these aggressive treatments, which cause significant morbidity and mortality, relapse is common and five-year overall survival is only 30–40%. Moreover, the majority of patients is over the age of 65 and are not candidates for high dose chemotherapy, leading to a five-year overall survival of 5–10% in this group [4,5].

Recent studies have demonstrated that AML is organized as a cellular hierarchy initiated and maintained by leukemia stem cells (LSC) which possess the canonical stem cell properties of self-renewal and the capacity to generate vast numbers of leukemic progenitors and blasts [6,7]. A key implication of this cancer stem cell model is that LSC must be eliminated for cure; however, LSC have demonstrated resistance to standard chemotherapy and radiation treatment [8,9]. Identification of cell surface molecules preferentially expressed on clinically relevant AML stem cells offers an attractive strategy for the development of novel AML therapies, as these cell surface molecules can serve as potential targets for monoclonal antibody therapy. A number of cell surface molecules preferentially expressed on AML LSC compared to normal human hematopoietic stem and progenitor cells have been identified, including CD47 [10].

CD47 possesses a single immunoglobulin variable region (IgV)-like extracellular domain and regulates multiple cellular processes implicated in immune responses [11]. It is widely expressed on hematopoietic and non-hematopoietic cells; however, we previously found that CD47 was more highly expressed on AML LSC than their normal counterparts, and that increased CD47 expression in AML is associated with poor clinical outcomes [6,7,12]. CD47 makes a number of protein-protein interactions including *in cis* with integrins and *in trans* with two ligands, thrombospondin-1 (TSP-1) and signal regulatory protein alpha (SIRPα). SIRPα encodes an Ig-superfamily receptor whose cytoplasmic region contains immunoreceptor tyrosine-based inhibition motifs (ITIMs) and is expressed on macrophages, dendritic cells, and neurons [13–18]. Upon binding CD47, SIRPα initiates an inhibitory signal transduction cascade via recruitment of the src homology-2 domain containing protein tyrosine phosphatases SHP-1 and SHP-2, which in turn deliver inhibitory signals for phagocytosis [19–22]. In normal physiology, CD47 was discovered to be an age marker on mouse RBCs, which exhibit progressively decreasing expression of CD47 likely leading to their eventual phagocytic removal by sinusoidal macrophages of the spleen, suggesting that the more aged RBCs are likely to be most at risk for extravascular phagocytosis by CD47 blocking antibodies [17,18,23].

The complex process of phagocytosis depends on the relative balance of pro-phagocytic and anti-phagocytic inputs [2]. Based on these observations, we proposed a model in which leukemia cells accumulate pro-phagocytic signals, many of which are not molecularly characterized. As a consequence, leukemia cells expressing high levels of CD47 are likely selected to counter pro-phagocytic signals. In this way, leukemia cells are dependent on CD47 expression to prevent phagocytic elimination by innate immune cells [24].

From this model, we predicted that blockade of the CD47-SIRPα interaction would result in dominance of pro-phagocytic signals resulting in phagocytosis of the leukemia cells. We validated this hypothesis by demonstrating that an available blocking mouse anti-human CD47 antibody, B6H12, stimulated phagocytosis and reduced the burden of AML engraftment in primary human xenograft models [6]. We also hypothesized that a blocking anti-CD47 antibody would synergize with a second antibody able to bind Fc-receptors and deliver a potent pro-phagocytic signal. Consistent with this idea, we found that B6H12 and rituximab potently synergized in the eradication of NHL in xenograft models [25]. Finally, CD47 expression was detected on cancer cells from many hematologic and solid tumors, and we found that B6H12 enabled the phagocytosis of primary human cancer cells in vitro, inhibited the growth of orthotopically xenotransplanted human tumors, and prevented the metastasis of human tumor cells [26–30].

Collectively, these studies suggest that a humanized blocking anti-CD47 antibody may be an effective anti-cancer therapeutic both as monotherapy and in combinations. In the present study, we report the development of a novel humanized anti-human CD47 antibody, designated Hu5F9-G4, generated by complementarity determining region (CDR) grafting onto a human IgG4 scaffold to minimize the recruitment of antibody Fc-dependent effector functions. Hu5F9-G4 induced potent macrophage-mediated phagocytosis of primary human AML cells in vitro and completely eradicated human AML in vivo, leading to long-term disease-free survival of patient-derived xenografts. Moreover, Hu5F9-G4 synergized with rituximab to eliminate NHL engraftment and cure xenografted mice. Finally, toxicokinetic studies in non-human primates showed that Hu5F9-G4 could be safely administered intravenously at doses able to achieve potentially therapeutic serum levels. Thus, Hu5F9-G4 is actively being developed for clinical trials in human AML and solid tumors.

## Materials and Methods

### Antibody generation

A cDNA fragment of human CD47 encoding the extracellular domain was cloned from a full-length human CD47 cDNA (Open Biosystems) and was fused to mouse Fc to generate a CD47/mFc fusion protein, which was used to immunize mice to produce monoclonal mouse anti-human CD47 antibodies. Hybridomas were generated using standard protocols. In brief, 4–6 week old Balb/c mice were immunized with purified recombinant huCD47/mFc fusion protein twice a week for a total of 4 weeks. Titers were assessed thereafter and the spleen cells were fused with SP2/0 cells. Hybridomas were selected and supernatants from the resulting clones were screened by enzyme linked immunosorbent assay (ELISA) and fluorescent activated cell sorting (FACS).

### Antibody V cloning and sequencing

The cloning strategy used here involved an initial RNA isolation from hybridoma cells (Qiagen). The cDNA sequences encoding the heavy and light chain variable regions of 5F9 monoclonal antibody were obtained using 5’ RACE-PCR techniques (Clontech) and were sequenced using standard DNA sequencing techniques.

### Molecular modeling and antibody humanization

Humanization of mouse anti-CD47 5F9 antibody was performed by installing CDR residues from mouse antibody onto a human germline framework (FR) [31]. Briefly, mouse 5F9 was humanized by judicious recruitment of corresponding CDR residues. Differences between mouse 5F9 and the human FR residues were individually modeled to investigate their possible influence on CDR conformation. Humanized VH and VL genes were synthesized by McLab (South San Francisco, CA).

### Cell transfection

293F cells were cultured under FreeStyle™ 293 Expression Medium (Invitrogen). Transient transfection was performed by co-transfection of expression vectors encoding antibody heavy chain and light chain using 293fectin transfection reagent (Invitrogen), according to the manufacturer’s instructions. Four to five days later, supernatants from the transfected cells were harvested and tested for antibody secretion by ELISA. Briefly, 96-well plates (Nunc, Roskilde, Denmark) were coated with 1 μg/ml goat anti-human Fc gamma antibody in phosphate-buffered saline (PBS) for 16 hr at 4°C. After blocking for 1 hr with 0.4% BSA in PBS at room temperature, isolated supernatants were added in 1/3 sequential dilutions, and incubated for 1 hr at room temperature. Plates were subsequently washed three times and incubated with HRP-conjugated goat anti-human kappa-specific antibody for 1 hr at room temperature. After washing, plates were developed with OPT. The reaction was stopped with 2M H_2_SO_4_, and OD was measured at 490 nM.

### Antibody purification and characterization

The culture supernatant was applied to protein A Sepharose columns (GE Healthcare). The column was washed with PBS, and protein was then eluted with eluting buffer (0.1 M sodium citrate buffer, pH 3.0). Collected fractions were neutralized with 1 M Tris pH 9.0. Finally, purified samples were dialyzed against PBS. Purity of the eluted antibody fraction was analyzed by sodium dodecyl sulfate polyacrylamide gel electrophoresis (SDS-PAGE) on 10% gels under reducing or non-reducing conditions. Bands were visualized by Coomassie brilliant blue staining.

### Cell surface antigen-binding assay

YB2/0 cells that had been stably transfected with human CD47 were incubated with 10 μg/ml of Hu5F9-G4 or HuIgG4 at 4°C for 1 hr. Then, the cells were washed with PBS three times, followed by incabation with 10 μg/ml PE-labeled anti-human Fc gamma specific antibody (eBiosciences, San Diego, CA, USA) at 4°C for 1 hr. Binding was measured using an FACSCanto (Becton–Dickinson, San Jose, CA, USA). Un-transfected YB2/0 cells were used as a negative control.

### Competition ELISA

A competitive binding assay was used to evaluate the antigen-binding specificity of the anti-CD47 mAbs. Briefly, 96-well plates (Nunc, Roskilde, Denmark) were coated with 1 μg/ml huCD47/mFc fusion protein in phosphate-buffered saline (PBS) for 16 hr at 4°C. After blocking for 1 hr with 0.4% BSA in PBS at room temperature, 10 μg/ml biotin-labeled mouse 5F9 was added into the plates in the presence of various concentrations of unlabeled Hu5F9-G4 at room temperature for 1 hr. Plates were subsequently washed three times and incubated with HRP-conjugated streptavidin (Thermo Scientific Pierce) for 1 hr at room temperature. After washing, plates were developed with OPT. The reaction was stopped with 2M H_2_SO_4_, and OD was measured at 490 nM.

### Antibody affinity measurement

A cynomolgus CD47 cDNA was cloned from cynomolgus spleen cDNA library (BioChain, China). The extracellular domain of cynomolgus CD47 was cloned and fused to mouse Fc to generate a cynoCD47/mFc fusion protein, which was used in the affinity measurement. Human CD47/mFc was made by fusing the extracellular domain of human CD47 cDNA (Open Biosystems) with mouse Fc as described above and used for measuring dimeric binding affinity to Hu5F9-G4. In addition, human CD47/his fusion protein was made by fusing the extracellular domain of human CD47 to His-tag and used for measuring monomeric binding affinity to Hu5F9-G4. Binding studies were done using a Biacore 2000 (GE). Running buffer was 10 mM HEPES pH 7.4, 150 mM NaCl, 0.005% tween-20. Data were collected at 25°C. Each mAb sample was amine coupled to a CM4 sensor chip using standard NHS/EDC activation (7 minutes). Each antibody was diluted to 10 ug/ml in 10 mM NaAcetate pH 5.0 and injected until the desired level of immobilization was obtained. Each surface was then blocked with 1 M ethanolamine for 7 minutes. Each sample was tested in duplicate.

### CD47-SIRPα interaction blocking assay

96-well plates (Nunc, Roskilde, Denmark) were coated with 1 μg/ml of SIRPα/human Fc fusion protein in phosphate-buffered saline (PBS) for 16 hr at 4°C. After blocking for 1 hr with 0.4% BSA in PBS at room temperature, 1μg/ml of CD47/mouse Fc protein was added either in the absence or presence of increasing concentrations of Hu5F9-G4 at room temperature for 1 hr. Plates were subsequently washed three times and incubated with an HRP-conjugated anti-mouse Fc secondary antibody for 1 hr at room temperature. After washing, plates were developed with OPT. The reaction was stopped with 2M H_2_SO_4_, and OD was measured at 490 nM. Each sample was tested in triplicates.

### *In vitro* phagocytosis assay

In vitro phagocytosis assays were performed as described previously. Briefly, HL-60 or primary human AML cells from different patients were CFSE-labeled and incubated with human peripheral blood-derived macrophages in the presence of different concentrations of mouse 5F9, Hu5F9-G4, or an isotype control antibody for 2 hr at 37°C. Cells were washed with IMDM serum-free media and resuspended in 200 μl media. Then, the cells were analyzed by fluorescence microscopy to determine the phagocytic index (number of cells ingested per 100 macrophages). Statistical analysis using Student's t test was performed with GraphPad Prism.

### Antibody-dependent cell-mediated cytotoxicity (ADCC) assay

ADCC was performed according to the instruction from aCella-TOX™ Bioluminescence Cytotoxicity kit (Cell Technology). In brief, human PBMC were incubated overnight at 10^6^ cells/mL in IMDM human complete medium with 400 unit/mL IL-2 (PeproTech, Rocky Hill, NJ, USA). The next day, activated PBMC were used as effector cells. 5 x10^3^ HL60 cells in 25 μL IMDM human complete medium were added per well in an U-bottom 96 well plate. Then, 25 μL of IMDM human complete medium containing various antibodies at the desired concentrations was added to each well. After 5 minutes incubation at 37°C, 2.5 x10^5^ PBMC in 25 μL IMDM human complete medium was added to each well to give a ratio of effector cells to target cells of 50:1. Mixtures were incubated at 37°C for 4 hours, followed by detection using reagents supplied by the kits. Assays were repeated three times and representative figures are shown here.

### Complement-dependent cytotoxicity (CDC) assay

5 x 10^4^ target cells in 50 μL IMDM + 10% FBS + Pen/Strep were added into each well of a 96-well U-bottom plate. 50 μL of antibodies was added at various concentrations (diluted with medium), starting from 10 μg/mL to 10^−5^ μg/mL, with 100 x dilution intervals. The mixture was incubated at room temperature for 5 minutes, and then 50 μL human complement serum was added to each well, mixed, and the plate was incubated at 37°C. After 2 hours incubation, 50 μL alamar blue (AbD Serotec) was added in each well, mixed, and the plate was incubated at 37°C overnight. On the next day, the plate was cooled to room temperature for 15 minutes and read by SpectraMax M3 plate reader for fluorescence signals at excitation wavelength of 530 nm and emission wavelength of 590 nm. Relative fluorescence unit (RFU) was use to indicate relative amount for live cells after assay. Assays were repeated three times and representative figures are shown here.

### CD47 receptor occupancy assay

CD47 binding standard curve on AML cells was made by using AF488-conjugated Hu5F9-G4 at various concentrations. Receptor occupancy was measured by incubating the target cells with unlabeled Hu5F9-G4 under different concentrations, and then the cells were either assayed in *in vitro* phagocytosis or incubated with a saturating concentration of AF488-Hu5F9-G4 based on the standard curve and analyzed for binding by flow cytometry. Receptor occupancy was calculated as follows:

$$\%\;\text{receptor occupancy}=100-\left({\text{MFI}}_{\text{test}}-{\text{MFI}}_{\text{unstained}\;/}{\text{MFI}}_{\text{saturated STD}}-{\text{MFI}}_{\text{unstained}}\right)\text{X}100$$

### Apoptosis assay

Human primary AML cells were thawed and viable mononuclear cells were isolated by gradient centrifugation over Ficoll using standard protocols. Viable cells were resuspended at 1x10^6^ per 50 ul in IMDM+5% FBS. 10 ug/ml Hu5F9-G4 or isotype control antibody was added and the cells were incubated at 37°C for 3 hr. Staurosporine was used as a positive control. Apoptotic cells were identified by staining with Annexin V (BD Biosciences, Cat. # 556547) according to the manufacturer’s instructions and analyzed by flow cytometry.

### *In vivo* antibody treatment of human AML engrafted mice

All mouse experiments were conducted according to an Institutional Animal Care and Use Committee–approved protocol (Stanford APLAC# 22264) and in adherence to the National Institutes of Health *Guide for the Care and Use of Laboratory Animals*. All the mice in this study were purchased from The Jackson Laboratory or bred in house. Any animal that exhibited weight loss, lethargy, hunched posture, or ruffled fur that did not improve within 1 week of treatment with Nutrical and fluids was euthanized. We euthanized animals when morbid per institutional protocols.

There were two groups of mice in the studies: the control group treated with mouse IgG and the experimental group treated with Hu5F9-G4 antibody. Deaths were expected in the control group due to bone marrow failure from fulminant leukemia. The experimental group survived well and was euthanized at the end of the study.

Cryopreserved cells from human sample SU028, SU048, and SU266 were thawed in 10 ml media (IMDM + 10% FBS + glutamax + penicillin streptomycin) and 100 μg of DNase I (StemCell) to perform T cell depletion. Briefly, cells were washed and resuspended in Robosep buffer. Human CD3 Positive Selection 18051-high recovery program was selected and after program completion, the CD3- fraction was collected, counted, and prepared for intravenous injection in HBSS (Life Technology) + 2% FBS buffer (Omega Science). Human primary AML xenograft model was established in NOD/SCID/IL-2Rgnull (NSG) female mice 8–10 weeks old. Mice were conditioned with 200 rads using a Faxitron CP-160 gamma irradiator up to 24 hours prior to intravenous injection. 100 μL of 5 x 10^6^ primary AML cells were then injected into NSG mice by tail vein with a Terumo 29 G needle. At 5-weeks post-transplant, bone marrow cells were aspirated from the femur using BD 27G needles and stained with mouse anti-mouse CD45.1 PECy7 (eBiosciences, San Diego, CA, USA), rat anti-mouse Ter119 PECy5 (eBiosciences, San Diego, CA, USA), mouse anti-human CD45 PB (BD Bioscience, San Jose, CA, USA), mouse anti-human CD3 APC-Cy7 (BD Bioscience, San Jose, CA, USA), mouse anti-human CD33 PE (BD Bioscience, San Jose, CA, USA), and mouse anti-human CD19 APC (BD Bioscience, San Jose, CA, USA). The percentage of human CD45+CD33+ AML cells was determined by flow cytometry. Based on the human AML cell engraftment level, five mice transplanted with each SU028 or SU048 sample were assigned into one of two treatment groups: mouse IgG control or Hu5F9-G4 at 100 μg daily by intraperitoneal injection. The treatment started at Day 1 and lasted for 14 consecutive days. Serum was collected at pretreatment and 2 hours post-treatment after the 1st, 5th, 8th, 12th, and 14th doses for PK analysis. Bone marrow cells were aspirated at different time points to analyze AML engraftment. A complete blood count was analyzed once a month using HemaTrue Hematology Analyzer. At Day 159, surviving mice were taken down for histology analysis of tibia bones. For the treatment of mice transplanted with SU266, four or five were assigned into one of three treatment groups: mouse IgG control, Hu5F9-G4 at 100 μg daily, and Hu5F9-G4 at 500 μg twice a week. The treatment started at Day 1 and lasted for 14 days. Serum was collected at pretreatment and 2 hours post-treatment for PK analysis. Bone marrow cells were aspirated at different time points to analyze AML engraftment. All mice were followed for overall survival, and the p-value of survival in Hu5F9-G4 treated groups was compared with the mouse IgG control groups. In the end of the study, mice were euthanized by the use of CO2 for rodent euthanasia.

### Synergy of Hu5F9-G4 and rituximab in Raji Luc-EGFP engrafted NSG mice

Mice were engrafted with Raji Luc-EGFP at a concentration at 1 million cells/mouse via tail vein injection. They were imaged *in vivo* to determine the level of engraftment seven days post engraftment. Treatment began the same day for 21 consecutive days. All mice were injected via intraperitoneal injection. Mice were imaged *in vivo* via IVIS Spectrum Imaging System at the following time points: Pre-treatment on Day 7 of engraftment, Post seven doses on Day 14 of engraftment, Post fourteen doses on Day 21 of engraftment, Post twenty-one doses on Day 28 and one week post-treatment on Day 35. Survival assessment was carried on Day 217. In the end of the study, mice were euthanized by the use of CO2 for rodent euthanasia.

### Toxicity study in non-human primates

All non-human primate experiments were conducted according to an Institutional Animal Care and Use Committee–approved protocol (Stanford APLAC# 26070) and in adherence to the National Institutes of Health *Guide for the Care and Use of Laboratory Animals*. The monkeys were socially-housed (update to 3 animals of the same sex and dosing group together) in stainless steel cages equipped with a stainless steel mesh floor and an automatic watering valve. All primary enclosures were in accordance with that as specified in the USDA Animal Welfare Act (9 CFR, Parts 1, 2 and 3) and as described in the Guide for the Care and Use of Laboratory Animals (National Research Council. Guide for the Care and Use of Laboratory Animals. Washington, DC: National Academy Press. Current edition). Each cage was clearly labeled with a cage label indicating study, group, animal number, and sex. Temperature: 64°F to 84°F (18°C to 29°C); Humidity: 30% to 70%; Light Cycle: 12 hours light and 12 hours dark (except during designated procedures); Ventilation: Ten or more air changes per hour with 100% fresh air (no recirculation).

For the dose-escalation study, two female animals were enrolled and dosed at one week intervals: one NHP was administered a single dose of EPO (17,000 U/kg) 5 days prior to the first dose of Hu5F9-G4 (3, 10, 30, 100, and 300 mg/kg), and one NHP was administered Hu5F9-G4 (1, 3, 10, 30, and 100 mg/kg) with no pre-treatment of EPO. Blood samples were collected for measurement of complete blood counts including hemoglobin and for Hu5F9-G4 serum level.

In separate NHP studies, female cynomolgus monkeys were administered a single 1-hour IV infusion of 0.1, 0.3, 1, 3, 10 or 30 mg/kg of Hu5F9-G4 or PBS vehicle as a control group (one animal per group) on Day 1, or a priming dose (PD) on Day 1 at doses of 1 or 3 mg/kg, followed by weekly maintenance doses (MD) of 10 or 30 mg/kg Hu5F9-G4 through Day 43. Samples collected in the different studies were as follows:

1. Hematology. Single dose: blood was withdrawn twice before the injections and at 3, 6, 10 and 14 days following the administration. PD/MD dose: blood was withdrawn twice before the injections and at 3, 6, 10, 13, 17, 20, 24, 27, 29, 36, and 43 days following the administration.
2. Clinical chemistry. Single dose: blood was withdrawn twice before the injections and at 6 and 14 days following the administration. PD/MD dose: blood was withdrawn twice before the injections and at 6, 13, 20, 29, 36, and 43 days following the administration.
3. Urinalysis. Single dose: up to 14 ml of urine was collected twice prior the injection and at 3 and 10 day after the injection. PD/MD dose: up to 14 ml of urine was collected twice prior the injection and at 3, 10, 17, and 24 day after the injection.
4. Pharmacokinetic analysis. Blood was collected by venipuncture into tubes with no anticoagulant at different time points as indicated in Fig 4B and 4D. Serum level of Hu5F9-G4 was measured by ELISA as described above using CD47/mouse Fc fusion protein as the coating reagent, followed by detection with an HRP-conjugated anti-human Kappa secondary antibody.

All animals were monitored twice daily for mortality and moribundity checks. Body weights were measured at least once pre-study and weekly thereafter starting on day 7.

## Results

### Generation of monoclonal antibodies against human CD47

**Fig 1:**
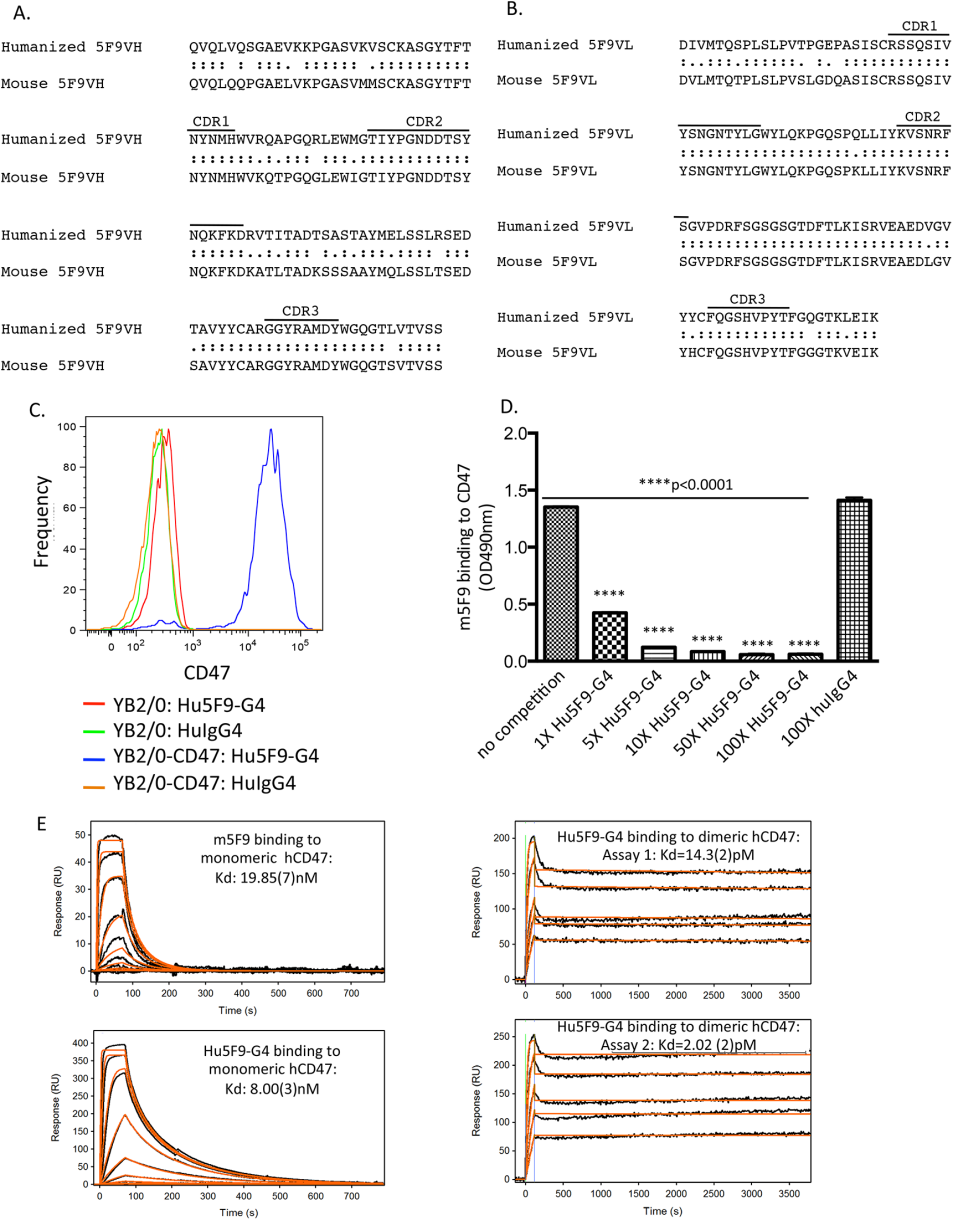
Cloning, humanization, and characterization of anti-human CD47 monoclonal antibody 5F9. (A-B) Comparison of mouse and humanized 5F9 variable heavy (A) and light (B) regions. CDRs are marked as indicated. (C) Rat YB2/0 cells stably transfected with human CD47 and parental YB2/0 cells were stained with human IgG4 isotype control or Hu5F9-G4 and analyzed for surface binding by flow cytometry. (D) Biotinylated mouse 5F9 binding to human CD47/mFc was detected by ELISA in the absence or presence of increasing concentrations of unlabeled Hu5F9-G4 or isotype control. Each sample was assayed in duplicate and SEM values are presented. Indicated p-values were determined by Two-Way ANOVA in comparison. (E) Binding of Hu5F9-G4 to monomeric or dimeric CD47 was analyzed by surface plasmon resonance yielding the indicated traces and binding constants. Data were processed by subtracting responses from the reference surface as well as an average of buffer injections. The responses were globally fit to a 1:1 interaction model including a term for mass transport. The number in parentheses represents the standard error in the last reported digit.

A cDNA fragment of human CD47 encoding the extracellular domain was fused to mouse Fc to generate a hCD47/mFc fusion protein, which was used to immunize mice to produce monoclonal mouse anti-human CD47 antibodies. The specificity of selected hybridoma clones was examined by FACS binding to either parental or human CD47-transfected rat YB2/0 cells. One of the positive clones was obtained and designated as 5F9. Using universal antibody primers, heavy and light chain variable regions of 5F9 were successfully cloned. Multiple clones of each V gene product were sequenced to monitor PCR-induced errors, and the amino acid sequences of VH and VL of 5F9 were determined (Fig 1A and 1B). DNA sequence analysis demonstrated that the heavy chain of 5F9 uses a V segment of the IGHV1 family, and that the light chain belongs to the IGKV1 subgroup.

### Molecular modeling and humanization of 5F9 antibody

In order to humanize 5F9, several 3D models of the Fv of the target antibody were built using combinations of known structures. 2PCP and 2JEL exhibiting 93% and 95% identity to 5F9, respectively, were used for the VL modeling. 2PCP and 1CIC exhibiting 70% and 78% identity to 5F9, respectively, were used for the VH modeling. To select human antibody framework regions (FR) to be used as templates for CDR-grafting, the mouse 5F9 VL and VH regions were compared with those of human germline sequences. The FRs of mouse 5F9 VL region were found to have the highest homology with the VK2-28 subgroup, and the FRs of the VH region exhibited the highest homology with the human VH1-3 subgroup. The FRs from human VK2-28 and VH1-3 were therefore used as the bases for designing humanized 5F9. Amino acid positions in the FR regions that differ between 5F9 and VK2-28/VH1-3 sequences, and that may influence antigen binding, were identified. Based on molecular modeling, identical and non-identical residues in the FRs were retained (Fig 1A and 1B).

We previously demonstrated that anti-human CD47 antibody activity is primarily dependent on blocking the CD47-SIRPα interaction [25]. Thus, in order to reduce the potential for toxicity against normal CD47-expressing cells, we constructed humanized 5F9 variable regions onto a human IgG4 scaffold (Hu5F9-G4) that recruits fewer Fc-dependent effector mechanisms compared to different human IgG subclasses. The human IgG4 heavy chain constant region was also modified to incorporate the Ser228Pro substitution to reduce the rate of Fab arm exchange that is a feature of human IgG4 molecules [32–34].

### Characterization of antigen binding activity of Hu5F9-G4

Antigen binding activity of Hu5F9-G4 was tested by flow cytometry using rat YB2/0 cells stably transfected with human CD47. Hu5F9-G4 bound to CD47-transfected YB2/0 cells, but not to parental YB2/0 cells (Fig 1C). To assess the antigen binding specificity of Hu5F9-G4, competition binding between humanized and parental mouse 5F9 was conducted by ELISA, and showed that Hu5F9-G4 competed mouse 5F9 for CD47 binding in a dose-dependent manner, while an isotype control antibody at the maximum concentration had no impact (Fig 1D). Thus, Hu5F9-G4 possesses the same antigen binding specificity as its parental antibody. We next measured the antigen binding affinity of Hu5F9-G4 using surface plasmon resonance. Hu5F9-G4 bound to monomeric human CD47 antigen with a K_D_ of 8x10^-9^ M, and bound to a bivalent human CD47 antigen with a K_D_ of 8x10^-12^ M (Fig 1E), well within the range of clinically approved antibodies.

### Hu5F9-G4 induces potent macrophage-mediated phagocytosis of AML

**Fig 2:**
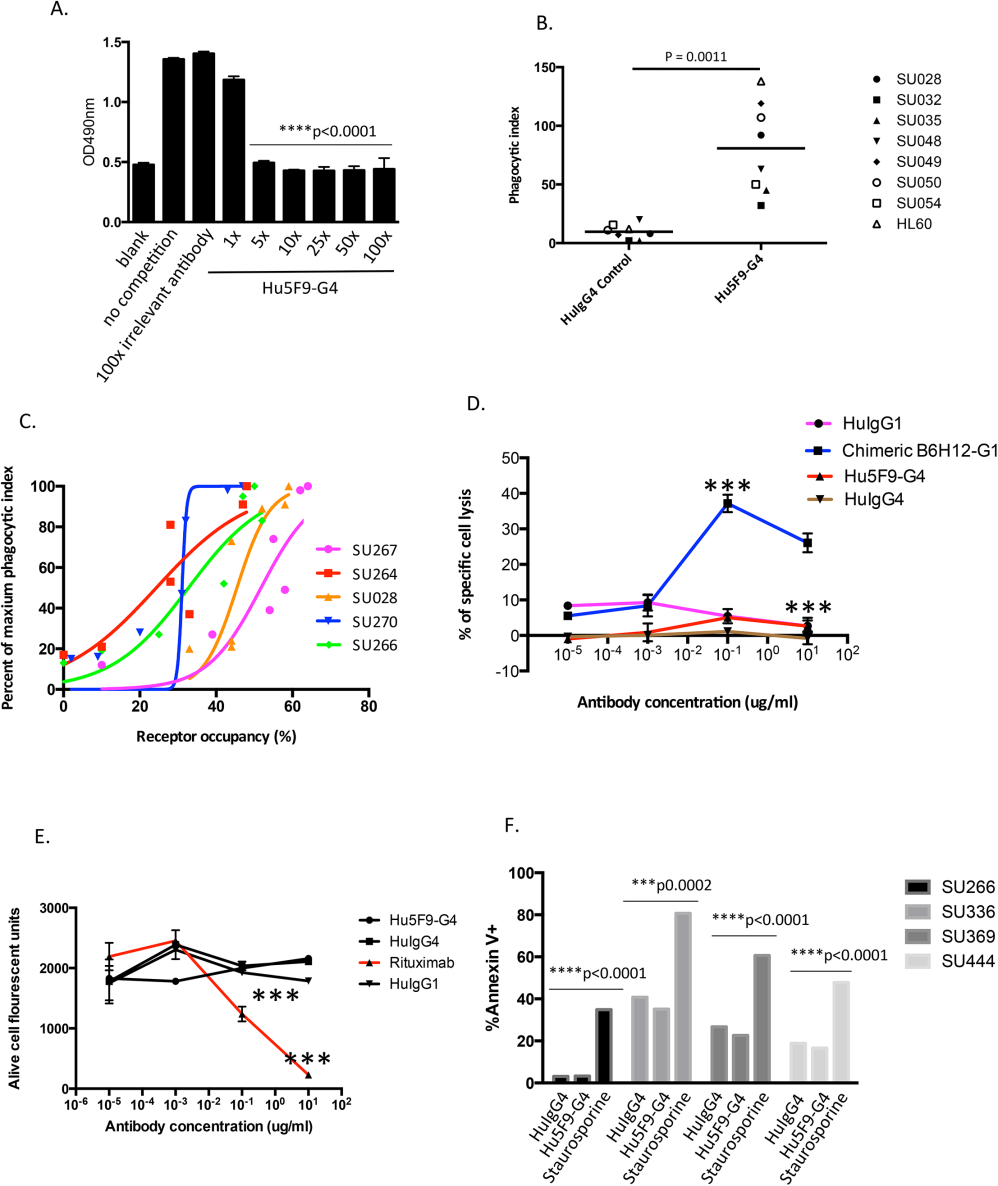
Hu5F9-G4 induces potent macrophage-mediated phagocytosis of AML. (A) Recombinant SIRPα/human Fc fusion was coated in 96-well plates and CD47/mouse Fc fusion protein was added either in the absence or presence of an equal amount, 5, 10, 25, 50 or 100-times more Hu5F9-G4. The binding activity of CD47 to SIRPα was measured by an HRP-conjugated anti-mouse Fc specific secondary antibody. Each sample was performed in triplicate and SEM values are presented. (B) HL-60 and seven primary human AML cells were labeled with CFSE and incubated with human peripheral blood-derived macrophages in the presence of 10 ug/ml Hu5F9-G4 or isotype control. Two hours later, macrophages were imaged by fluorescence microscopy to determine the phagocytic index (number of target cells ingested per 100 macrophages) in duplicate. Lines indicate mean values and the indicated p-value was determined using a two-sided t-test. (C) The relationship between CD47 receptor occupancy and phagocytosis was determined with primary human AML cells. AML cells were first incubated with increasing concentrations of unlabeled Hu5F9-G4 (0.01–10 ug/ml), and after 2 hours, free CD47 receptor was measured using a saturating concentration (40 ug/ml) of Alexa 488-labeled Hu5F9-G4 followed by flow cytometry. In parallel, the AML cells were evaluated for susceptibility to phagocytosis via addition of human peripheral blood-derived macrophages and determination of the phagocytic index for each condition. (D) HL-60 cells were incubated with increasing concentrations of the indicated isotype control antibodies, chimeric B6H12-IgG1 as a positive control, or Hu5F9-G4, and ADCC activity was determined with human PBMC effector cells. The experiment was repeated 3 times. *** p<0.0001 as determined by t-test. (E) SUDHL4 cells were incubated with increasing concentrations of the indicated isotype control antibodies, rituximab as a positive control, or Hu5F9-G4 and CDC activity was determined with human complement-containing serum. The experiment was repeated 3 times. *p <0.0001 as determined by t-test. (F) Four human primary AML cell samples were incubated with 10 ug/ml Hu5F9-G4 for 3 hours. Apoptotic cells were identified by staining with Annexin V followed by flow cytometry analysis. Human IgG4 isotype control antibody and Staurosporine were used as negative and positive controls, respectively.

We next tested the ability of Hu5F9-G4 in blocking the interaction between CD47 and SIRPα. We coated 96-well plates with recombinant SIRPα and detected binding of CD47-mouse Fc fusion protein with an anti-mouse secondary antibody. As shown in Fig 2A, CD47 bound SIRPα in the presence of an irrelevant control antibody, however, the binding activity was blocked when Hu5F9-G4 was added. We then investigated whether Hu5F9-G4 blockade of the CD47/ SIRPα interaction enabled phagocytosis of target cells. The HL-60 AML cell line and primary human AML cells (S1 Table) were used as the target cells and labeled with CFSE. Phagocytic activity was measured by using human peripheral blood-derived macrophages and counting the number of ingested cells using fluorescence microscopy. Hu5F9-G4 induced potent phagocytosis of HL-60 cells and all seven different human primary AML samples tested (Fig 2B). In addition, Hu5F9-G4 efficiently enabled phagocytosis of HL-60 cells at levels comparable to the native mouse 5F9 antibody across a range of concentrations tested (data not shown).

In order to determine the degree of CD47 receptor occupancy necessary to trigger phagocytosis, primary human AML cells (S1 Table) were incubated with Hu5F9-G4 at different concentrations. After two hours, free CD47 receptor was measured using fluorophore-labeled Hu5F9-G4 and flow cytometry. The primary human AML cells were then evaluated for susceptibility to phagocytosis via addition of human peripheral blood-derived macrophages. The data indicated that a range of 40–60% CD47 receptor occupancy was required for maximal phagocytosis of AML cells *in vitro* (Fig 2C).

Hu5F9-G4 was engineered on a human IgG4 scaffold to minimize recruitment of Fc-dependent effector functions such as ADCC and CDC. The ability of Hu5F9-G4 to induce ADCC activity was tested against HL-60 cells. As a positive control, chimeric B6H12-IgG1 (ChB6H12-G1), an anti-CD47 antibody with a human IgG1 constant region, mediated ADCC in a dose-dependent manner. In contrast, Hu5F9-G4 did not induce ADCC at any of the concentrations tested (Fig 2D). CDC activity of Hu5F9-G4 was tested against SUDHL4, an NHL cell line expressing both CD20 and CD47. As a positive control, the anti-CD20 antibody rituximab mediated CDC activity in a dose-dependent manner. In contrast, Hu5F9-G4 was unable to induce CDC (Fig 2E).

Antibodies directed against CD47 have been shown to directly induce apoptosis of several malignant hematopoietic cell lines when immobilized or cross-linked [35–38]. Unlike treatment with staurosporine, Hu5F9-G4 did not induce apoptosis of human primary AML cells (Fig 2F) or HL-60 cells (data not shown), consistent with our prior observation that B6H12 did not induce apoptosis of primary AML cells [6]. Collectively, these in vitro studies indicate that the mechanism of action of Hu5F9-G4 does not involve ADCC, CDC, or apoptosis, but rather activation of macrophage-mediated phagocytosis.

### Hu5F9-G4 antibody eradicates primary human AML *in vivo*

**Fig 3:**
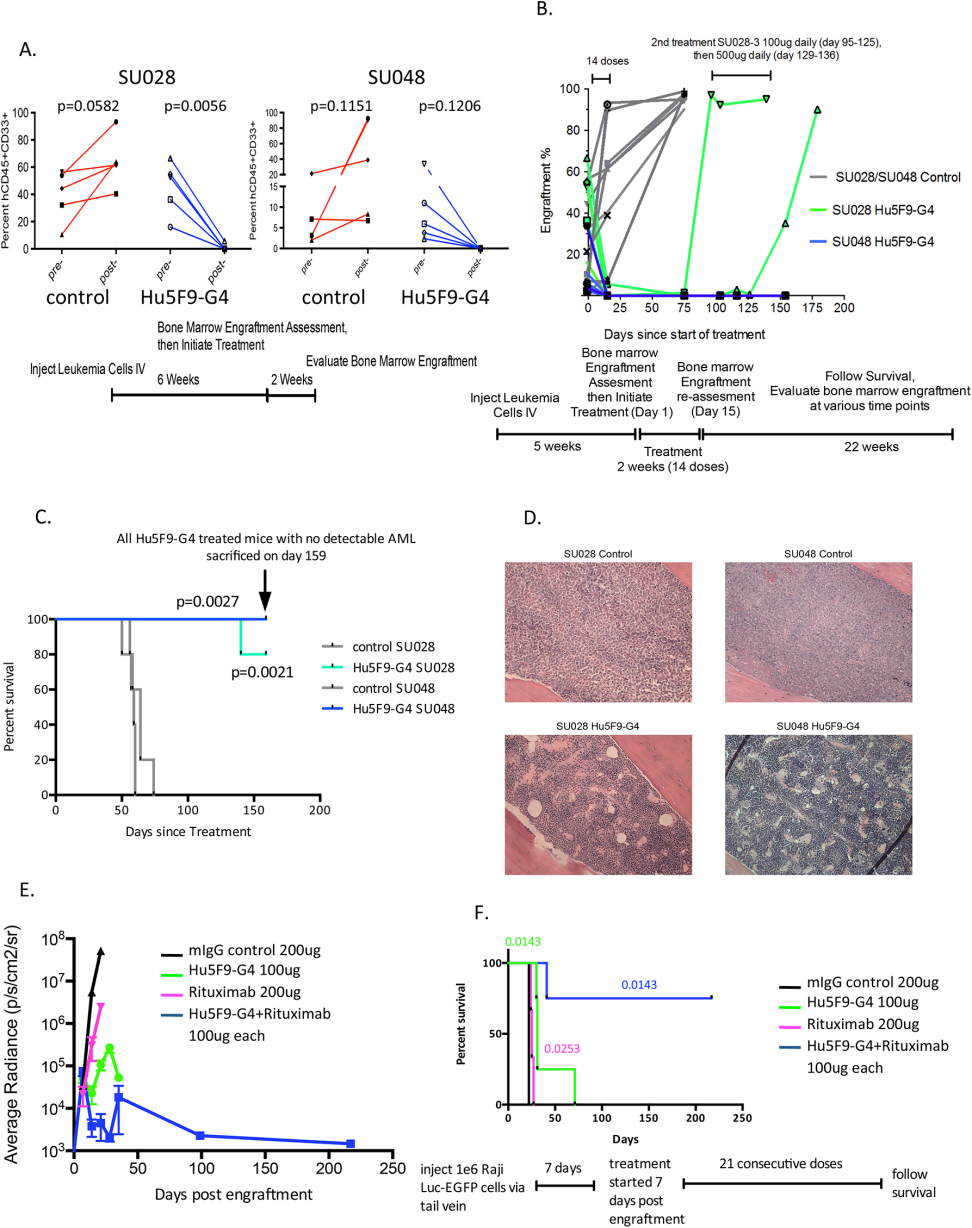
Hu5F9-G4 eradicates primary human AML and synergizes with rituximab to eliminate lymphoma. (A) Primary human AML cells (5e6) from samples SU028 and SU048 were engrafted into 10 NSG mice per the indicated scheme. Engrafted mice were assigned to treatment with daily injections of either control IgG or Hu5F9-G4 for 2 weeks (n = 5 mice per group). The percent of hCD45+CD33+ leukemic blasts in the bone marrow was determined pre- and post-treatment in all mice by flow cytometry. The indicated p-values were determined by paired t-test. (B) At the end of treatment, mice were monitored for an additional 22 weeks with periodic assessment of human leukemic engraftment in the bone marrow. For the two SU028 mice with recurrence of disease, additional treatment and response is indicated. (C) Kaplan-Meier plot of overall survival of SU048 and SU028 cohorts treated with control IgG or Hu5F9-G4 is indicated. The indicated p-values were derived by log rank test. All mice treated with Hu5F9-G4 were sacrificed on Day 159 for histology and bone marrow analysis. (D) H&E sections of representative mouse bone marrow from mice engrafted with SU028 or SU048 post-treatment with either control IgG or Hu5F9-G4 antibody. (E) Luciferase-labeled Raji cells were transplanted intravenously into NSG mice as a model of disseminated lymphoma. 7 days after transplantation, mice were assigned to daily treatment with control IgG (200 ug), Hu5F9-G4 (100 ug), rituximab (200 ug), or combination of Hu5F9-G4 (100 ug) and rituximab (100 ug) for 21 days. Mice were imaged repeatedly up to more than 200 days to determine the bioluminescent radiance. Relative to the IgG control, p-value of tumor growth inhibition is 0.0013, 0.0065, and 0.0013 on day 21 in Hu5F9-G4-, rituximab-, and combination-treated groups, respectively. Relative to Hu5F9-G4- and rituximab-treated groups, p-value of tumor growth inhibition is 0.0112 and 0.0003 on day 21 in combination-treated groups, respectively. On day 28, p-value of tumor growth inhibition is 0.0059 in Hu5F9-G4 and combination-treated groups. P-values were determined by t-test. (F) Kaplan-Meier plot of overall survival of Raji-engrafted cohorts from panel E. The indicated p-values were derived by log rank test.

To investigate the ability of Hu5F9-G4 to eliminate primary human AML *in vivo*, we employed an established disease model in which AML cells from 2 primary patient samples, SU048 and SU028, were transplanted into immunodeficient NSG mice and allowed to engraft robustly in the bone marrow prior to starting therapy. Mice with 2–67% engraftment in the bone marrow as measured by bone marrow biopsy and FACS analysis were administered daily intraperitoneal injections of 100 μg of either Hu5F9-G4 or a control antibody for 2 weeks. Analysis of the bone marrow at the end of treatment showed a significant reduction in leukemic burden in mice treated with Hu5F9-G4, while leukemic engraftment increased in control IgG-treated mice (Fig 3A). Most importantly, Hu5F9-G4 completely eradicated AML cells in the SU048-engrafted mice with no recurrence over an additional 22 weeks of monitoring (Fig 3B). Hu5F9-G4 also initially cleared the bone marrow in SU028-engrafted mice; however, 2 out of 5 mice with higher leukemia burden prior to treatment recurred several weeks later with near complete effacement of the mouse bone marrow by human leukemia cells. At this point, further treatment with Hu5F9-G4 failed to clear the AML (Fig 3B). In total, 8 out of 10 Hu5F9-G4 treated SU028- and SU048-engrafted mice survived for 159 days without recurrence of AML (Fig 3C). At Day 159, these mice were sacrificed for H&E histologic analysis of the bone marrow, which confirmed that human AML cells were completely eliminated and that normal mouse hematopoiesis had recovered (Fig 3D). Consistent with this, complete blood counts including white blood cells, hemoglobin, and platelets also returned to the normal range (S2 Table). Taken together, these in vivo studies demonstrated that Hu5F9-G4 eliminated primary human AML engraftment and profoundly improved long-term disease-free survival.

We next sought to determine the serum levels of Hu5F9-G4 associated with the efficacy observed in this study. Daily 100 ug dosing for 14 days resulted in Hu5F9-G4 serum levels maintained in the range of 100–200 ug/ml (S1 Fig). We conducted additional studies varying the Hu5F9-G4 dose and schedule and determined that sustained serum levels in the range of 50–150 ug/ml are necessary to achieve optimal anti-leukemic activity (data not shown).

Based on these studies, we investigated the activity of Hu5F9-G4 against another primary human AML sample, SU266, in which the mice were treated with Hu5F9-G4 using different dosing regimes. As before, mice were transplanted with SU266 leukemic cells and monitored for engraftment in the bone marrow. 16 weeks later, bone marrow leukemic burden ranged from approximately 1–80% as measured by bone marrow biopsy and FACS for human and mouse cells. Mice with similar levels of disease were assigned to 3 treatment groups: control IgG, Hu5F9-G4 100 ug per day, and Hu5F9-G4 500 ug twice a week for 2 weeks. As expected, all control IgG-treated mice exhibited a higher burden of disease at the end of treatment and eventually died (S2A and S2B Fig). 4 mice treated with daily 100 ug doses of Hu5F9-G4, and 4 treated with twice weekly 500 ug doses, completely cleared the bone marrow and experienced no recurrence resulting in long-term disease-free survival. Thus, several dosing regimens demonstrated in multiple primary human AML cases that Hu5F9-G4 cleared the bone marrow and significantly extended overall disease-free survival.

### Hu5F9-G4 synergizes with rituximab to eliminate lymphoma engraftment

We have previously reported that anti-CD47 antibody (clone B6H12) synergized with rituximab to eliminate Raji cells in a disseminated NHL model. Here we investigated if similar synergy could be achieved with Hu5F9-G4. Mice engrafted with luciferase-labeled Raji cells were assigned to treatment with either control IgG (200 ug), Hu5F9-G4 (100 ug), rituximab (200 ug), or the combination of Hu5F9-G4 and rituximab, and then followed by *in vivo* bioluminescent imaging to determine the level of engraftment (Fig 3E), and for overall survival (Fig 3F). Control mice exhibited rapid lymphoma dissemination and were euthanized on Day 22 due to morbidity. Hu5F9-G4 or rituximab monotherapy showed a modest survival benefit compared to control, but did not cure any mice. However, combination treatment of Hu5F9-G4 with rituximab completely eliminated disease in 3 of 4 mice leading to extended overall disease-free survival and demonstrating that Hu5F9-G4 potently synergizes with rituximab in the treatment of NHL-engrafted mice.

### Non-human primate pharmacokinetic and toxicology studies show no serious adverse events associated with Hu5F9-G4

Hu5F9-G4 was developed from a mouse anti-human CD47 antibody that lacks binding to mouse CD47. In order to identify a relevant animal model for pharmacokinetic and toxicology assessments, we investigated CD47 from cynomolgus monkeys (cyno). Cyno CD47 differs from human CD47 at only 3 amino acids in its extracellular domain, none of which are present in the CD47-SIRP-alpha interaction interface [39]. We engineered a cyno-CD47 Fc fusion protein, and found that Hu5F9-G4 bound cyno CD47 with an affinity of 10x10^-12^ M based on surface plasmon resonance measurements, which was very similar to its affinity for human CD47 (S3A Fig). In addition, Hu5F9-G4 bound cynomolgus monkey CD47, but not mouse, by an enzyme-linked immunosorbent assay (ELISA) (S3B Fig). Finally, Hu5F9-G4 bound CD47 on the surface of cyno leukocytes and red blood cells, similar to its binding to human cells (data not shown).

**Fig 4:**
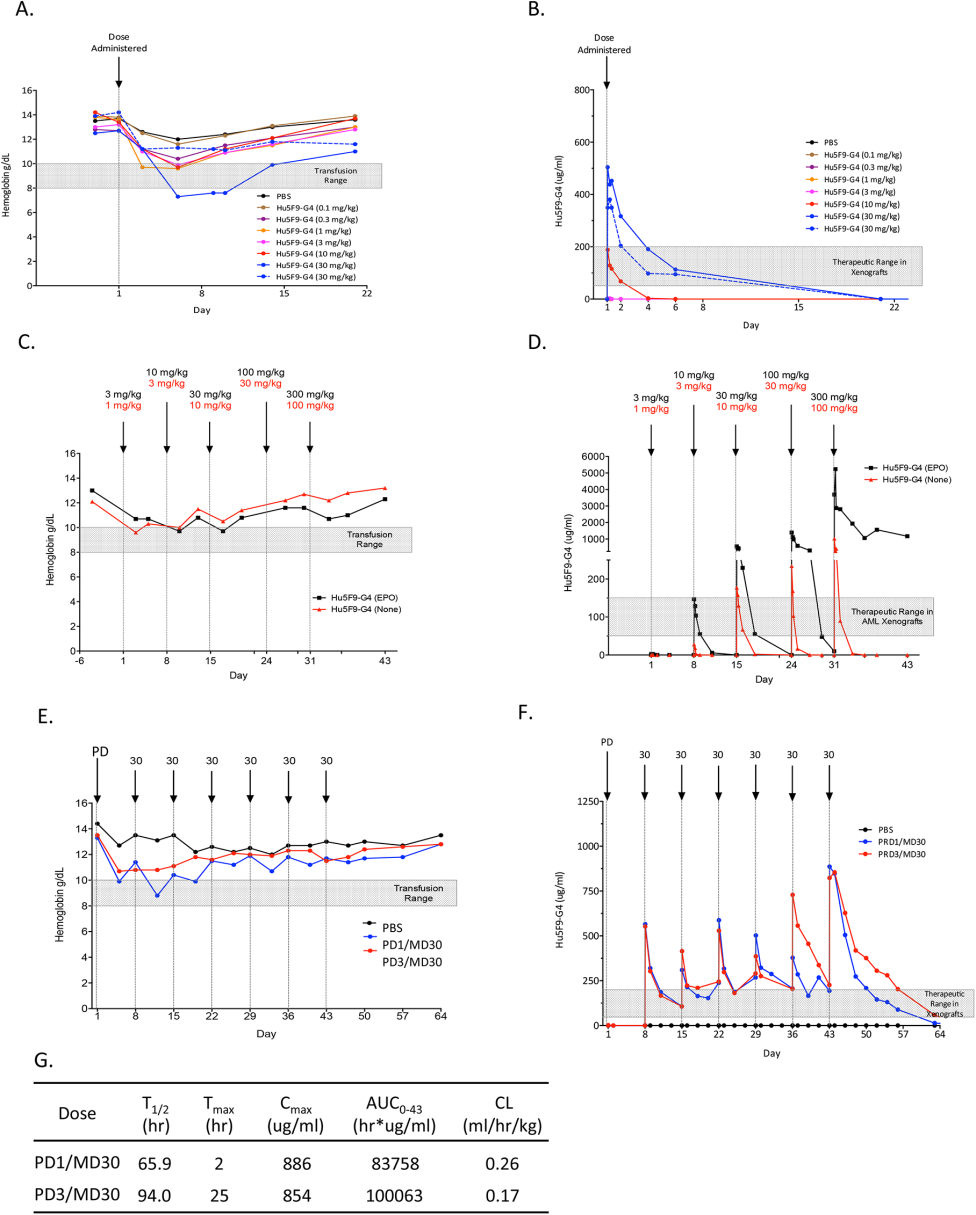
Non-human primate pharmacokinetic and toxicology studies show no serious adverse events associated with Hu5F9-G4. (A) Individual cynomolgus monkeys were administered single intravenous infusions of Hu5F9-G4 at the indicated doses. The hemoglobin level was monitored over 3 weeks. The shaded bar indicates the range of hemoglobin that might trigger transfusion in humans. (B) Serum Hu5F9-G4 levels were determined from the monkeys dosed in panel A. The shaded bar indicates the range of serum Hu5F9-G4 associated with efficacy against human cancer in xenograft studies. (C) Individual cynomolgus monkeys that received either no pre-treatment (red) or pre-treatment with a single dose of EPO (black) were administered Hu5F9-G4 in a dose escalation study with the doses and time points indicated. Hemoglobin was serially measured to monitor anemia. The shaded bar indicates the range of hemoglobin in humans that might trigger transfusion. (D) Serum Hu5F9-G4 levels were determined from the monkeys dosed in panel C. The shaded bar indicates the range of serum Hu5F9-G4 associated with potent efficacy against primary human AML in xenograft studies. (E) Cynomolgus monkeys were administered a priming dose (PD) on Day 1 of either 1 or 3 mg/kg, followed by once weekly maintenance doses (MD) of 30 mg/kg at the indicated time points. Hemoglobin was serially measured to monitor anemia, and the shaded bar indicates the range of hemoglobin in humans that might require transfusion. (F) Serum Hu5F9-G4 levels were determined from the monkeys dosed in panel E; the shaded bar indicates the range of serum Hu5F9-G4 associated with efficacy against human cancer in xenograft studies. (G) Pharmacokinetic parameters in cynomolgus monkeys dosed in panel E. T_1/2_: half-life, T_max_: time of C_max_, C_max_: maximum observed concentration, AUC: area under the curve, CL: clearance.

We first assessed Hu5F9-G4 administered to cynomolgus monkeys as a single intravenous infusion at 0, 0.1, 0.3, 1, 3, 10, and 30 mg/kg in separate individuals. All animals were evaluated for changes in clinical signs, food consumption, body weights, and clinical pathology parameters. Administration of Hu5F9-G4 was generally well tolerated, and no treatment-related effects were noted on a comprehensive list of clinical observations, food consumption, body weights, or clinical chemistry parameters indicative of renal, hepatic, or cardiac effects. Clinical hematology assessment indicated that Hu5F9-G4 caused a dose-dependent anemia associated with reticulocytosis and spherocytosis in all animals. The nadir of the anemia occurred approximately 5–7 days after the infusion and generally correlated with dose (Fig 4A). Note that the severity of the anemia was variable as the two animals administered 30 mg/kg exhibited different responses. Importantly, in all animals the anemia spontaneously resolved, returning to baseline levels after approximately two weeks (Fig 4A). In all cases, no free plasma hemoglobin was detected, indicating the absence of intravascular hemolysis. No other abnormalities of white blood cells or platelets were observed. Thus, consistent with its known function in regulating RBC phagocytosis, Hu5F9-G4 caused a transient anemia, likely due to erythrophagocytosis, but was otherwise well-tolerated.

With single-dose administration of Hu5F9-G4, pharmacokinetic data demonstrated that only the 10 and 30 mg/kg dose levels were able to transiently achieve serum levels in the range associated with efficacy in xenograft studies (Fig 4B). This is likely due to the large antigen sink of CD47 expressed on circulating red and white blood cells, in addition to other non-hematopoietic tissues.

Based on the previously described role of CD47 in the normal clearance of aging red blood cells [17], the Hu5F9-G4-related anemia observed in this study was considered related to the pharmacological action of Hu5F9-G4 binding to CD47 expressed on RBCs. The premature loss of RBCs was compensated by an ensuing reticulocytosis, and over time, the initial anemia resolved with replacement by younger cells. Moreover, we speculated that prior administration of erythropoietin (EPO) would blunt the anemia by stimulating young RBC production.

From these considerations, we conducted a separate dose-escalation study in NHP based on the hypothesis that initial low doses would blunt the loss of RBC and stimulate production of less-susceptible young RBC, thereby facilitating tolerance of subsequent larger doses (Fig 4C). Two animals were enrolled into this study and dosed at one-week intervals: one with EPO pre-treatment (3, 10, 30, 100, and 300 mg/kg), and one with no pre-treatment (1, 3, 10, 30, and 100 mg/kg). In both cases, the NHP exhibited a mild anemia with initial dosing that did not worsen with repeated administrations. In fact, the hemoglobin only reached the upper threshold for transfusion in humans, even without EPO pretreatment. Strikingly, the animals tolerated all doses well, including 100 and 300 mg/kg, with no additional blood or metabolic abnormalities. At the end of the study, both animals were euthanized, and necropsy and histopathology analysis revealed no abnormalities.

From this dose-escalation study, we determined the pharmacokinetics of Hu5F9-G4 in NHP (Fig 4D). Consistent with the presence of a large antigen sink of CD47 expressed by normal tissues, the initial low doses of Hu5F9-G4 were rapidly cleared from the serum. In contrast, the higher doses of Hu5F9-G4 produced sustained serum levels indicating saturation of the antigen sink. Remarkably, the animal dosed at 300 mg/kg had a peak level of 5 mg/ml with a sustained level of more than 1 mg/ml for at least 2 weeks. These data, suggest that a priming dose followed by a larger maintenance dose regimen should be capable of achieving the sustained 50–150 ug/ml serum level that was associated with potent efficacy in the pre-clinical xenograft models.

These results led us to conduct another NHP pilot study using a priming-maintenance dosing approach to model potential clinical dosing strategies. The goal of the priming dose would be to stimulate production of young RBCs that would then facilitate larger maintenance doses capable of achieving sustained serum levels. We conducted a study in cynomolgus monkeys in which a priming dose (PD) of either 1 or 3 mg/kg was administered on Day 1, followed one week later by weekly maintenance dosing (MD) of 30 mg/kg for six weeks. All animals were evaluated for changes in clinical observations, food consumption, body weights, and clinical pathology parameters. No mortalities or changes in key clinical chemistry parameters indicative of renal, hepatic, or cardiac effects were noted. Administration of Hu5F9-G4 was well tolerated over the entire dosing course. In both cases, the priming dose resulted in mild anemia and reticulocytosis. As hypothesized, the maintenance doses were well-tolerated with no further declines in hemoglobin throughout the treatment course (Fig 4E). By the end of the study, hemoglobin levels returned to the normal range. Pharmacokinetic analysis indicated that exposure to Hu5F9-G4 as measured by C_max_ and the area under the serum concentration curve (AUC_0–43_) in both animals achieved sustained serum Hu5F9-G4 levels within or above the potential therapeutic range for the duration of the maintenance dosing period with prolonged half-life after the final dose (Fig 4F and 4G). These results suggest that PD1/MD30 or PD3/MD30 dosing strategies saturate the CD47 antigen sink. Collectively, these cynomolgus monkey studies demonstrated that a low priming dose of Hu5F9-G4 results in a modest anemia and compensatory reticulocytosis response that enabled subsequent higher maintenance doses of drug to be well tolerated.

## Discussion

CD47 is a critical regulator of phagocytosis that is expressed on leukemia and cancer cells where it functions to inhibit phagocytosis by cells of the innate immune system. We previously demonstrated that blocking monoclonal antibodies against human CD47 enable the phagocytic elimination of leukemia cells and cancer cells from many human solid tumors. Here, we describe the generation and pre-clinical assessment of a novel humanized anti-CD47 antibody, Hu5F9-G4, for efficacy against AML and NHL, as well as toxicokinetic properties in non-human primates. Hu5F9-G4 binds human CD47 with tight affinity, blocks the CD47-SIRPα interaction, enables phagocytosis of primary AML cells in vitro, eliminates human AML in xenograft models, synergizes with rituximab in the eradication of NHL xenografts, and was safely administered intravenously to non-human primates at doses able to achieve serum levels much higher than the potential therapeutic range. Thus, Hu5F9-G4 is actively being developed for clinical trials in human AML and solid tumors.

Monoclonal antibodies are a class of cancer therapeutic agents that demonstrate specificity for their antigenic targets and can recruit Fc-dependent effector functions. The use of mouse monoclonal antibodies in clinical settings is limited by the development of human anti-mouse immune responses that can impact both safety and pharmacokinetic properties of the drug. We generated a novel mouse antibody, 5F9, that specifically binds human CD47 and enables phagocytosis of human cancer cells. To develop 5F9 into a candidate human therapeutic antibody, humanization was conducted by grafting mouse CDRs of the variable regions onto human framework sequences resulting in Hu5F9-G4. Hu5F9-G4 possesses similar antigen binding specificity as its parental antibody 5F9, and it binds to CD47 with an affinity well within the range of clinically approved efficacious antibodies. Anti-CD47 antibodies may facilitate elimination of tumor cells through a variety of mechanisms [40,41]. It has been reported that CD47 antibodies enhance nonphagocytic tumor cell killing by neutrophils and NK cells [42,43]. In this study, we demonstrated that Hu5F9-G4 blocks the CD47-SIRPα interaction and potently enables the phagocytosis of leukemia cells (Fig 2A–2C). No ADCC, CDC, or direct apoptosis of target cells was induced by Hu5F9-G4 (Fig 2D–2F). However, we can not rule out the possibility that Hu5F9-G4 may induce phagocytosis in an Fc-dependent manner, as human macrophages have been described to express the high-affinity Fc receptor FcRγI [44]. As with our prior studies, we determined that Hu5F9-G4 synergizes with rituximab in the elimination of NHL in xenograft models (Fig 3E and 3F). This potent effect likely involves a pro-phagocytic signal delivered by the human IgG1 Fc of rituximab through Fc-receptors on phagocytes coupled with blockade of the anti-phagocytic CD47 signal by Hu5F9-G4. We anticipate that Hu5F9-G4 will synergize with any antibody capable of engaging Fc-receptors to deliver a pro-phagocytic signal including trastuzumab, cetuximab, and many other antibodies for oncologic indications.

Pharmacodynamic markers monitor biological effects and are used in pre-clinical and clinical studies for establishing doses and dosing regimens for future studies. For the clinical development of Hu5F9-G4, we developed a human CD47 receptor occupancy assay as a potential predictive marker of response to Hu5F9-G4 antibody therapy. Using this assay, we observed a relationship between the percent receptor occupancy and Hu5F9-G4-induced phagocytic activity for multiple primary human AML samples. In all cases, optimal anti-tumor activity of Hu5F9-G4 could be achieved without full receptor occupancy (Fig 2C), with maximal activity occurring with receptor occupancy ranging between 40–60%. Notably, different AML patient samples exhibited markedly distinct receptor occupancy curves illustrating significant heterogeneity in response to Hu5F9-G4. Although the determinants of this heterogeneity are unknown, we did observe different CD47 expression levels in the primary AML cells (data not shown). Nevertheless, different cells may have different thresholds between CD47 receptor occupancy and phagocytosis.

We show here that Hu5F9-G4 can effectively eliminate AML and AML LSC *in vivo*, even in cases with a significant burden of bone marrow disease. However, we did observe several mice in which treatment of Hu5F9-G4 was eventually followed by relapsed disease that did not respond to further Hu5F9-G4 therapy. To investigate potential mechanisms involved in this lack of further response, we isolated leukemic cells from these mice and assessed them in vitro. These leukemic cells were still partly bound by Hu5F9-G4 and were able to be phagocytosed by human macrophages (data not shown). Thus, it appears that expression of CD47 and Hu5F9-G4 binding epitopes were retained on these cells; moreover, pro-phagocytic signals were still present. We speculate that the likely cause of the poor responses upon retreatment is due to the very high level of disease in the bone marrow (>95% at the time of retreatment) that may make it impossible for the residual bone marrow resident macrophages to eliminate the leukemia cells. Consistent with this, transplantation of these relapsed leukemia cells into secondary recipients found them to be responsive to Hu5F9-G4 when treatment was initiated with a lower bone marrow burden of disease (data not shown). Thus, these relapsed cells were neither CD47 loss variants or immunoselected anti-CD47 resistant cells.

CD47 was discovered to be an age marker on mouse RBCs, which exhibit progressively decreasing expression of CD47 likely leading to their eventual phagocytic removal by sinusoidal macrophages of the spleen, liver, and bone marrow, suggesting that RBCs are likely to be most at risk for extravascular phagocytosis by CD47 blocking antibodies [17,18,23]. Indeed, in the single dose toxicity study in non-human primates, we observed a dose-dependent decrease in RBC, hemoglobin, and hematocrit starting on day 2, and reaching a nadir between days 5 and 7 (Fig 4A). The severity of the anemia was generally mild although significant variability was observed. This anemia was accompanied by a reticulocytosis that led to complete recovery by 2–3 weeks. Significantly, there was no free plasma hemoglobin detected in any of the animals, indicating an absence of intravascular hemolysis. We speculated that the anemia observed in the single-dose pilot study was related to the known role of CD47 in the normal clearance of aging red blood cells. As previously reported, RBCs gradually lose CD47 expression as they age, lose sialic acids from glycoproteins and glycolipids, and reorganize membrane phospholipids in a manner that presumably accumulates prophagocytic signals, leading to their elimination by phagocytosis [45]. However, a recent study showed that CD47 appeared to undergo a conformational change in experimental aging of erythrocytes that may switch the molecule from an inhibitory signal into an activating signal [46]. We showed here that by blocking CD47, Hu5F9-G4 leads to the premature loss of RBCs that is compensated for by an ensuing reticulocytosis. The initial anemia resolves as RBCs are replaced with younger cells and the age distribution of the red blood cell pool is shifted to younger cells that are presumably more resistant to the effects of CD47 blockade. Thus, we developed several dosing strategies to mitigate the initial anemia. First, we conducted a dose-escalation study in NHP with or without EPO pre-treatment. In both cases, NHP exhibited a mild anemia with initial low doses of Hu5F9-G4 that did not worsen with repeated higher doses, and the NHPs tolerated doses up to 100 and 300 mg/kg without abnormalities. Remarkably, the animal dosed at 300 mg/kg achieved a peak level of 5 mg/ml with a sustained level of more than 1 mg/ml for at least 2 weeks. We did notice that EPO treatment seemed to affect the PK (Fig 4D). However, this was a pilot study with only one animal, and studies of more animals (or eventually humans) will be necessary to further investigate this effect. We postulated that administration of an initial low priming dose of Hu5F9-G4 would stimulate a mild anemia and accompanying reticulocytosis that would facilitate subsequent higher maintenance doses of Hu5F9-G4 that were capable of achieving pharmacokinetic targets. Based on these considerations, a second pilot study was conducted in which monkeys were administered a priming dose of either 1 or 3 mg/ kg followed by weekly maintenance doses at 30 mg/kg. As predicted, the priming dose resulted in an initial modest anemia, which was not worsened by subsequent maintenance doses (Fig 4E) that were able to exceed PK targets (Fig 4F and 4G). This dosing strategy will likely need to be supplemented by RBC transfusions in those patients with AML who lack the ability to mount an effective reticulocytosis response to anemia. It remains to be seen how transfused RBCs will react to Hu5F9-G4 in vivo.

The immunogenicity of biopharmaceuticals used in clinical practice remains a challenge in drug development. Non-human primates are the most frequently used relevant animal model for the development of monoclonal antibodies, but the immune response of NHPs to therapeutic monoclonal antibodies is not considered to be predictive of the response in humans because of species differences. However, the presence of anti-drug antibody (ADA) may impact on PK profiles, safety, and drug exposure [47]. ADA responses should be further investigated in subsequent GLP (good laboratory practice) IND (Investigational New Drug)-enabling pharmacology and toxicology studies.

In conclusion, we have developed Hu5F9-G4, a humanized blocking anti-CD47 antibody that exerts anti-leukemic effects both in vitro and in vivo leading to the eradication of AML in xenotransplant mouse models and a major survival benefit. Significantly, toxicokinetic studies of Hu5F9-G4 in non-human primates showed that Hu5F9-G4 could be safely administered intravenously at doses able to achieve potentially therapeutic serum levels, using a priming-maintenance dosing strategy. These studies provide the pre-clinical rationale for the development of Hu5F9-G4 as a therapeutic either as a single agent or in combination with other cancer-targeting antibodies.

## Supporting Information

S1 Fig Pharmacokinetic analysis of Hu5F9-G4 serum levels in AML-engrafted mice treated with Hu5F9-G4. (PDF)

S2 Fig Hu5F9-G4 eradicates SU266 primary human AML engraftment *in vivo*. (PDF)

S3 Fig Hu5F9-G4 binds to cynomolgus monkey CD47. (PDF)

S1 Materials and Methods (DOC)

S1 Table Clinical and molecular characteristics of primary human AML samples manipulated in vitro and/or in vivo. (DOC)

S2 Table Complete blood count analysis of AML-engrafted mice after treatment with either control IgG (B) or Hu5F9-G4 antibody (A). (DOC)
