# Supplementary figures and images for: Pre-Clinical Development of a Humanized Anti-CD47 Antibody with Anti-Cancer Therapeutic Potential
Source: PLoS One. 2015 Sep 21;10(9):e0137345. doi: 10.1371/journal.pone.0137345 (PMC4577081; doi:10.1371/journal.pone.0137345)

Supplementary Figure 1

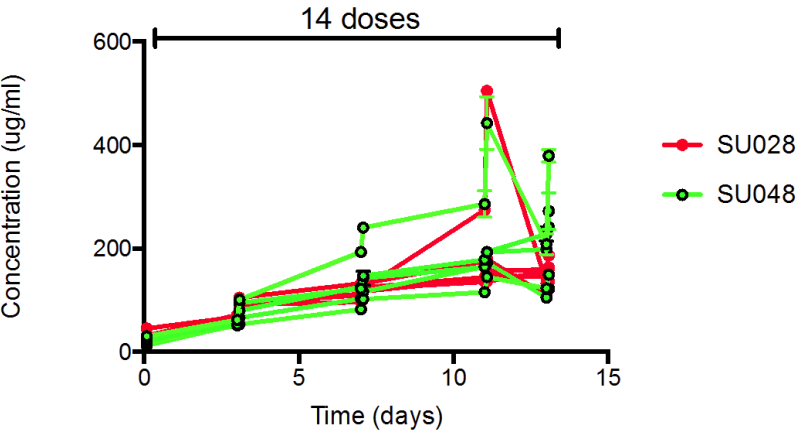

Supplement: S1 Fig — (PDF) [file pone.0137345.s001.pdf]

## Supplementary Figure 2

A.

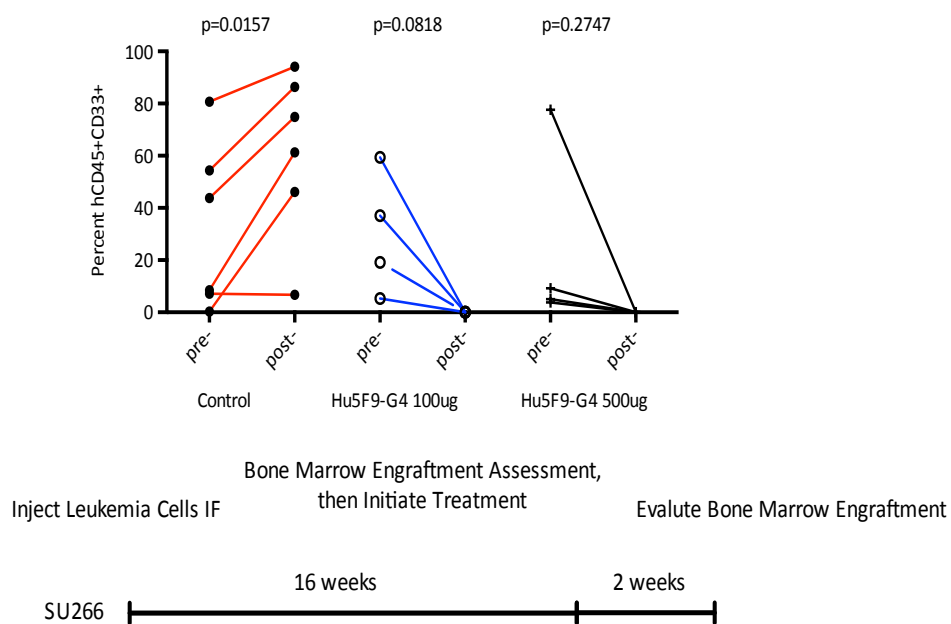

B.

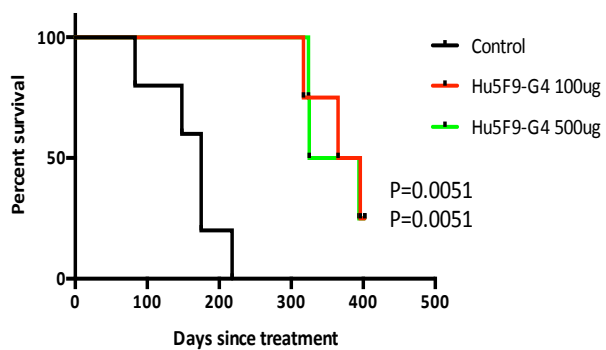

Supplement: S2 Fig — (PDF) [file pone.0137345.s002.pdf]

Supplementary Figure 3

A

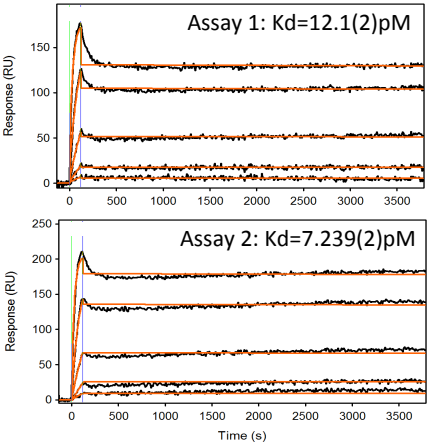

B

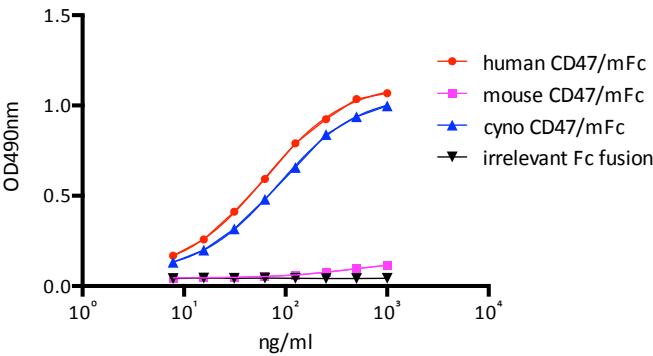

Supplement: S3 Fig — (PDF) [file pone.0137345.s003.pdf]
