# Supplementary material for: Pre-Clinical Development of a Humanized Anti-CD47 Antibody with Anti-Cancer Therapeutic Potential
Source: PLoS One. 2015 Sep 21;10(9):e0137345. doi: 10.1371/journal.pone.0137345 (PMC4577081; doi:10.1371/journal.pone.0137345)
Supplement: S1 Materials and Methods — (DOC) [file pone.0137345.s004.doc]

Supplementary Materials and Methods

**Animal Care**

Inhalational CO2 in a dedicated chamber was used for euthanasia in the in vivo mice study per institutional protocols as outlined below:

I. Characteristics

Carbon dioxide (CO2) is a safe and humane method of euthanasia that is a preferred technique for use with adult rodents, when properly used. The gas is inexpensive, nonflammable, and non-explosive. Furthermore, several rodents can be euthanized simultaneously. It causes no accumulation of exogenous chemical residues in tissues nor does it produce observable histological changes in non-pulmonary tissues (however
there may be slight pulmonary effects). It can be administered using inexpensive equipment that can be located anywhere in a facility or fixed to a mobile platform for portable use. Exposure to high concentrations of CO2 has an initial rapid depressant and anesthetic effect, which is followed by death through asphyxiation. However, high concentrations of CO2 may be distressful to some species. Accordingly, the chamber should not be pre-filled when euthanizing rats and mice. Experts maintain that a slow fill rate of 10-30% of the chamber volume per minute is the best welfare compromise between speed of onset, and nociception. It is recommended that 100% CO2 be used. Under these conditions, an exposure time 8-10 minutes at 100% CO2 appears generally adequate to euthanize adult animals. To shorten the time needed to perform euthanasia, sublethal exposure to CO2 can be followed by exsanguination, cervical dislocation or
decapitation. Carbon dioxide should be purchased in compressed gas cylinders. CO2 generated from other sources, such as dry ice, fire extinguishers, or from Alka-Seltzer®, are unacceptable because gas flow cannot be regulated precisely.

II. Humane Considerations

The most common errors when using CO2 for euthanasia are: (1) not using a gradual displacement rate of 10-30%; (2) overcrowding animals in the chamber; (3) not cleaning the chamber between sequential use; and (4) not euthanizing animals in their home cage.
Animals placed together in chambers must be of the same species, and, if needed, should be restrained so that they will not hurt themselves or other animals. Chambers must not be overcrowded. Overcrowding of the euthanasia chamber has been noted to lead to inadequate narcosis and asphyxiation. The number of animals should be limited to allow free flow of CO2 to each animal. Animals must be able to keep all four feet on the floor of the euthanasia chamber, must be able to turn around and must not be so crowded that they must sit on top of each other other (i.e., ideally <5 mice in a standard size mouse cage, but no more than 10). In this regard, it is important to also consider that mixing unfamiliar or incompatible animals (i.e., animals from different cages, especially males) in the same container may be distressful. If you do mix animals from different cages in the same euthanasia chamber, they must be euthanized immediately to minimize any
potential for aggression or distress. Chambers should be kept clean to minimize odors that might distress animals subsequently euthanized.

III. Methods

Compressed Gas

Use of a compressed gas cylinder to administer CO2 should always include an appropriate two-stage regulator and gas flow meter. The animal is placed inside the chamber and the lid secured. A fill rate of 10-30% of the chamber volume per minute with CO2, added to existing room air in the chamber should be appropriate to achieve the objective of rapid unconsciousness with minimal distress to the animals. The
flow rate can be increased once the animals have lost consciousness, however, animals should be closely observed during the filling process, as individual systems may require adjustment to achieve the desired effect.

IV. Removal from Chamber

Death is verified after euthanasia and prior to disposal. Since the anesthetic effects of CO2 are reversible, animals that are prematurely removed from the chamber prior to death can recover. Unintended recovery after the procedure will be very rare if appropriate CO2 concentrations and exposure times are used. However, whenever an inhalant gas is used for euthanasia, it is imperative to be sure that all animals
are dead when removed from the chamber. Before animals are removed, all visible movement (including breathing) should have stopped. After removal, check again to confirm the absence of multiple vital signs (loss of bladder control, absence
of heart rate, lack of a toe-pinch response, cessation of respiration). Confirmation of death should not be based solely on a single sign, for example, cessation of respiration. CO2 narcosis can be followed with a secondary method of euthanasia, cervical dislocation. After having been outside of the chamber and exposed to room air for at least 5-10 minutes and have been carefully observed for multiple vital signs, animals were disposed of in a black plastic bag that was labeled with the protocol number.

We monitored the health of the engrafted mice three times a week. When the mouse in the mouse IgG treated control group exhibits weight loss, lethargy, hunched posture, or ruffled fur, we monitored every day in order to record the survival days and collect tissues for data analysis.

In order to analyze AML engraftment in both control and experimental mice, we collected bone marrow cells by intrafemoral bone marrow aspiration. To minimize animal suffering and distress, adult mice (>6 weeks of age) were anesthetized by isofluorane inhalation (2% isofluorane in 100% oxygen at a flow rate of 2L/min) administered by nose cone to reach a steady state of anesthesia, determined by toe pinch reflex and slow steady breathing. Prior to the procedure, mice were administered carprofen 5 mg/kg SQ to minimize pain and 0.5-1.0 ml warm 0.9% saline for supportive care. During the procedure, the knee joint was disinfected with a surgical scrub (Nolvasan or Betadine Surgical Scrub) alternating with 70% EtOH. A sterile 27-gauge needle with attached syringe containing 20 microliters of phosphate buffered saline with anti-coagulant was inserted just under the patellar tendon so that the needle was lodged securely between the two condyles of the femur. Then the needle swiveled outward and upward so that it was parallel with the shaft of the femur. This action prevents the direct puncture of the patellar tendon and potential discomfort for the animal. The needle was then turned clockwise and counter-clockwise while being advanced slowly in the femoral marrow cavity. The needle is inserted just until there was a noticeable reduction in resistance. The correct positioning of the needle was confirmed by gently moving the syringe laterally with resistance from the interior surface confirming that the needle had been correctly placed in the femoral cavity. Bone marrow was aspirated by gently withdrawing the plunger on the syringe yielding 20-50 microliters of mouse bone marrow. Then the needle was fully removed from the femur. After the procedure, mice were removed from the nose cone and placed on a clean paper towel to prevent aspiration of bedding during recovery. All mice were monitored to verify recovery from anesthesia prior to placing back on the mouse rack. The procedure was completed when anesthetized mice had recovered and were ambulating normally in the cage. Mice were carefully monitored for signs of obvious and debilitating lameness in addition of weight loss, lethargy, hunched posture, or ruffled fur.

Non-human primate animal enrichment as described below:

All animals were socialized to provide for psychological enrichment. Animals of the same sex were socialized within dose groups after initial compatibility tests were completed. If an animal showed signs of health concerns (e.g., distress, body weight loss) or aggression, the animals would be separated at any time during the study (this was not an issue for this study, however). As a reward and means to promote operant conditioning and desired behavior, each animal was offered a food treat following study-related procedures as deemed necessary throughout the duration of the study. During dosing, the animal were fed food treats and/or given oral electrolyte solutions (e.g., Gatorade).

Veterinary Care:

Veterinary care was available throughout the course of the study and animals were examined by the veterinary staff as warranted by clinical signs or other changes. All veterinary examinations and recommended therapeutic treatments, if any, were documented in the study records. In the event that animals showed signs of illness or distress, the responsible veterinarian would make an initial recommendations about treatment of the animal(s) and/or alteration of study procedures, which must be approved by the Study Director. All such actions were properly documented in the study records and, when appropriate, by protocol amendment.

Treatment of the animal(s) for minor injuries or ailments could be approved without prior consultation with the Sponsor representative when such treatment does not impact fulfillment of the study objectives. If the condition of the animal(s) warranted significant therapeutic intervention or alterations in study procedures, the Sponsor representative was contacted, when possible, to discuss appropriate action. If the condition of the animal(s) is such that emergency measures had be taken, the Study Director and/or attending veterinarian would attempt to consult with the Sponsor representative prior to treatment, but the Study Director and/or veterinarian had authority to act immediately to alleviate suffering. The Sponsor representative was fully informed of any such events.

Animals were euthanized by intravenous injection of a commercially available veterinary euthanasia solution (documented in study records) followed by exsanguination. Clinical signs were measured by morning and evening cageside observations for any abnormal behavior, body weight, food consumption, physical examinations, ophthalmology examinations, clinical pathology (clinical chemistry, hematology, coagulation, urinalysis), macroscopic examination, organ weights, and microscopic examination of tissues.
