# Supplementary material for: Pre-Clinical Development of a Humanized Anti-CD47 Antibody with Anti-Cancer Therapeutic Potential
Source: PLoS One. 2015 Sep 21;10(9):e0137345. doi: 10.1371/journal.pone.0137345 (PMC4577081; doi:10.1371/journal.pone.0137345)
Supplement: Supplementary file 3 [file pone.0137345.s005.doc]

Supplementary Table 1

Sample	Age	Gender	Primary/ Secondary	De Novo/ Relapsed	Cytogenetics	Flt-3 ITD	%CD34	WHO Classification	FAB
SU028	53	Female	Primary	Relapsed	Complex	Positive	50	AML not-otherwisw specified	M5
SU032	47	male	Primary	De Novo	Normal	Negative	68	AML not-otherwisw specified	M5
SU035	46	male	Primary	De Novo	failed to grow	Negative	98	AML not-otherwisw specified	M5
SU048	75	Female	Primary	De Novo	Unknown	Positive	1	AML not-otherwisw specified	unknown
SU049	55	male	Primary	De Novo	Complex	Negative	<1	AML with 11q23 (MLL)	M5
SU050	75	male	Primary	Relapsed	Normal	Positive	<1	AML with multilineage dysplasia without antecedent MDS	M5
SU054	64	male	Primary	Relapsed	Normal	Positive	40	AML with multilineage dysplasia following MDS	unknown
SU266	64	male	Primary	De Novo	inv(3)	negative	96	AML with inv(3) (q21q26)	unknown
