# Supplementary material for: Pre-Clinical Development of a Humanized Anti-CD47 Antibody with Anti-Cancer Therapeutic Potential
Source: PLoS One. 2015 Sep 21;10(9):e0137345. doi: 10.1371/journal.pone.0137345 (PMC4577081; doi:10.1371/journal.pone.0137345)
Supplement: Supplementary file 4 [file pone.0137345.s006.doc]

Supplementary Table 2

A

Mouse	WBC (Day 144) normal range: 2.6-10.1 (10^3/ul)	HGB (Day 144) normal range: 10-16.1 (g/dl)	PLT (Day 144) normal range: 250-1540 (10^3/ul)
SU028 Hu5F9-G4-1	5.3	13.1	437
SU028 Hu5F9-G4-2	10.3	13.2	277
SU028 Hu5F9-G4-3	3.3	12.8	256
SU028 Hu5F9-G4-4	3.5	11.1	340
SU028 Hu5F9-G4-5	7.9	14.4	176
SU048 Hu5F9-G4-1	6.8	13	325
SU048 Hu5F9-G4-2	12.6	13.6	238
SU048 Hu5F9-G4-3	7.2	13	274
SU048 Hu5F9-G4-4	6.3	13.6	123
SU048 Hu5F9-G4-5	5.9	12.9	144

B

Mouse	WBC (Day 56) normal range: 2.6-10.1 (10^3/ul)	HGB (Day 56) normal range: 10-16.1 (g/dl)	PLT (Day 56) normal range: 250-1540 (10^3/ul)
SU028 Control-1	3.5	2.4	39
SU028 Control-2	6.9	7.3	208
SU028 Control-3	10.8	5.1	73
SU028 Control-4	11.8	6.6	153
SU028 Control-5	7.6	7.3	160
SU048 Control-1	17.8	15.1	351
SU048 Control-2	45.1	16.5	223
SU048 Control-3	26.3	12.8	215
SU048 Control-4	20.2	12.1	163
SU048 Control-5	64.6	12.9	171
